# Targeting the Biophysical Properties of the Myeloma Initiating Cell Niches: A Pharmaceutical Synergism Analysis Using Multi-Scale Agent-Based Modeling

**DOI:** 10.1371/journal.pone.0085059

**Published:** 2014-01-27

**Authors:** Jing Su, Le Zhang, Wen Zhang, Dong Song Choi, Jianguo Wen, Beini Jiang, Chung-Che Chang, Xiaobo Zhou

**Affiliations:** 1 Department of Radiology, The Wake Forest School of Medicine, Winston-Salem, North Carolina, United States of America; 2 College of Computer and Information Science, Southwest University, Chongqing, People's Republic of China; 3 School of Medicine and Dentistry, University of Rochester Medical Center, Rochester, New York, United States of America; 4 Jan and Dan Duncan Neurological Research Institute, Baylor College of Medicine, Houston, Texas, United States of America; 5 Department of Pathology, The Methodist Hospital Research Institute, Weil Cornell Medical College, Houston, Texas, United States of America; 6 Department of Mathematical Sciences, Michigan Technological University, Houghton, Michigan, United States of America; 7 Department of Pathology, Florida Hospital, University of Central Florida, Orlando, Florida, United States of America; Università degli Studi di Firenze, Italy

## Abstract

Multiple myeloma, the second most common hematological cancer, is currently incurable due to refractory disease relapse and development of multiple drug resistance. We and others recently established the biophysical model that myeloma initiating (stem) cells (MICs) trigger the stiffening of their niches *via* SDF-1/CXCR4 paracrine; The stiffened niches then promote the colonogenesis of MICs and protect them from drug treatment. In this work we examined *in silico* the pharmaceutical potential of targeting MIC niche stiffness to facilitate cytotoxic chemotherapies. We first established a multi-scale agent-based model using the Markov Chain Monte Carlo approach to recapitulate the niche stiffness centric, pro-oncogenetic positive feedback loop between MICs and myeloma-associated bone marrow stromal cells (MBMSCs), and investigated the effects of such intercellular chemo-physical communications on myeloma development. Then we used AMD3100 (to interrupt the interactions between MICs and their stroma) and Bortezomib (a recently developed novel therapeutic agent) as representative drugs to examine if the biophysical properties of myeloma niches are drugable. Results showed that our model recaptured the key experimental observation that the MBMSCs were more sensitive to SDF-1 secreted by MICs, and provided stiffer niches for these initiating cells and promoted their proliferation and drug resistance. Drug synergism analysis suggested that AMD3100 treatment undermined the capability of MICs to modulate the bone marrow microenvironment, and thus re-sensitized myeloma to Bortezomib treatments. This work is also the first attempt to virtually visualize in 3D the dynamics of the bone marrow stiffness during myeloma development. In summary, we established a multi-scale model to facilitate the translation of the niche-stiffness centric myeloma model as well as experimental observations to possible clinical applications. We concluded that targeting the biophysical properties of stem cell niches is of high clinical potential since it may re-sensitize tumor initiating cells to chemotherapies and reduce risks of cancer relapse.

## Introduction

Multiple myeloma (MM) and other tumors have a small population of tumor initiating (stem) cells that retain key stem cell properties including self-renewal and tumorigenesis [1]–[13]. Recent reports [3], [4] showed that a small population of CD138-negative B cells with “side population” characteristics present in myeloma. These cells have clonogenic potential *in vitro* and, when engrafted into immunodeficienct/nonobese diabetes (SCID/NOD) mice, can initiate de novo myeloma lesions of bulk of CD138+ cells in both primary and secondary transplant assays. Additionally, these myeloma initiating cells (MICs) have shown higher resistance to chemotherapeutic agents and thus are more likely to survive despite therapies [1]–[10]. These findings have led to the hypothesis that MICs survive chemo- and radio- therapies, regenerate the bulk of tumors, and thus cause the disease relapse. This idea is consistent with the clinical observation that disease relapse in multiple myeloma patients is common even if patients are treated with new therapeutic agents that can initially result in complete clinical responses [14]–[16]. Understanding and controlling MIC drug resistance is critical to the development of new therapies for the cure of myeloma.

Our group pioneered the research of the roles of biophysical properties in blood cancers and established the mechanism of the MIC-stroma positive feedback loop [17], [18]. Previous studies on the interactions between BMSCs and myeloma cells, especially MICs, have predominantly focused on biochemical communications such as the stimuli of growth factors, cytokines and chemotactic *via* paracrine signaling [19]. However, recent studies in solid tumors have indicated that a critical stage of the malignant transformation journey of cancer cells involves marked alterations in the biomechanical phenotype of the cell and its surrounding microenvironment [20], [21]. Indeed, it has been suggested that targeting the microenvironments (the “niches”) of the tumor stem cell could result in a reduction of the tumor burden [22]–[24]. Bone marrow stromal cells (BMSCs), one of the major cellular components in the MIC niches, are in close contact with MICs, and the biomechanical properties of BMSCs, besides chemical communications, also influence the local microenvironment of MICs and hence MIC fates. We have recently demonstrated that Myeloma-associated BMSCs (MBMSCs) from patients are much “stiffer” (higher Young's modulus level) and more contractile than Normal BMSCs (NBMSCs). Hydrogels are widely used to mimic the *in vivo* cellular microenvironments [25], [26], so we have utilized hydrogels of various stiffness levels to investigate the impact of such biophysical property on MIC-driven myeloma development. We have shown that stiffer hydrogels support colony formation and adherence of MICs better than softer hydrogels, suggesting that myeloma BMSCs provide myeloma cell-friendly microenvironments for MICs *via* exerting biomechanical forces [17], [18]. We also have demonstrated that MICs over-secrete SDF1 than mature myeloma cells and that treatment of CXCR4 inhibitor, AMD3100, leads to decreased adherence of MICs to MBMSCs, undermined colony formation potential of MICs, and better *in vitro* and *in vivo* drug efficacy of Bortezomib(BZM). These discoveries were also consistent with other reports [27].

With the perspective that the biophysical properties of MIC niches in bone marrow may be a promising drug candidate, there is an urgent need to develop mathematical models and tools to estimate drug effects on MICs within the context of their niches during when screening and evaluating drug candidates. Currently pharmaco-industries use the killing efficiency of the bulk tumor as the major *in vitro* drug screening criterion. Such practices overlook the importance of MICs cell microenvironments contributing to the common failure of translating promising drug candidates discovered in screening into clinical usage. However, mathematical models that involve the cancer initiating (stem) cells and their niches are still rare, except for few examples [28]–[30] including our recent work [31].

Modeling the dynamics of the MIC-derived myeloma lineage is crucial for associating the abnormality at cellular level with the features of the myeloma pathogenesis and pathophysiology at tissue level. We recently developed a myeloma lineage model [31] to systematically analyze the myeloma lineage development and experimentally monitor the major sub-populations in the lineage. Briefly, cells involved in the myeloma lineage were classified as myeloma initiating cells (MICs), myeloma progenitor cells (PCs), and matured myeloma cells (MMs). These three sub-populations were experimentally distinguishable by dual-staining for the expression of plasma cell surface markers CD138 and for side population (SP) using Hoechst staining, each showed unique features related with lineage sub-populations. MICs, recognized by SP staining and negative CD138 staining (SP/CD138−), showed unlimited renewal capability, potential to initiate and fully re-establish the whole myeloma lineage, and enhanced drug resistance. PCs, defined as the non-SP and negative CD138 expression (non-SP/CD138−) population, were only able to passage for limited rounds before beginning to express CD138 and losing the key PC character. The CD138+ sub-population, which was of 90% to 95% of the total myeloma population, was classified as the MMs and exhibit very limited proliferation capability. In this work we used similar definitions except further distinguishing terminal MMs (TMMs) that could no longer proliferate from those that still could divide (MMs).

To comprehensively illustrate the interaction between the myeloma initiating cells and their niches and to develop effective drug treatment, the following key questions need special attentions: (1) How do BMSCs, MICs, myeloma progenitor cells (PC), matured myeloma cells (MMs) and terminal MMs (TMMs) communicate in MIC niches? (2) How do the biomechanical phenotypes of BMSC, in terms of cell stiffness and contractibility, modulate MIC's growth and fates? (3) How do MIC growth and differentiation drive the development of myeloma? (4) How do the molecular level intracellular features of MICs and their stromal counterpart, BMSCs, contribute to the tissue level 3D cancer growth *via* the intercellular cell-to-cell interactions? And (5) How does the interaction between tumor cells and their niches change the drug treatment effect?

To address these questions, in this study we developed a 3D multi-scale agent-based model (ABM) using Markov Chain Monte Carlo approaches [32] to study the role of tumor–stroma interactions in multiple myeloma tumor progress. The system was classified into three levels: the intracellular, the intercellular, and the tissue level. Their relations were conceptually defined as “interfaces” among these levels. The agent-based model integrates events of multiple spatial and temporal scales. (1) Spatial scales: Intracellular signaling pathways were encapsulated into each cell type to determine the BMSC intercellular biomechanical phenotype (cell stiffness) or tumor cell behaviors (migration or proliferation). Cancer cells competed for the best location in 3D extracellular matrix to migrate or proliferate regarding to the change of BMSC cell's stiffness and sensitivity of chemoattractant (SDF1). In turn, chemoattractant cues at the tissue level triggered intracellular signaling pathways inside cells *via* the agent interfaces (receptors), and the resultant feedbacks were the changes of either cells' properties (change of cell stiffness or sensitivity to outside stimuli) or behaviors (secretion of chemoattractant, proliferation, differentiation, apoptosis, or migration). (2) Temporal scales: The model covered minute-to-hour-level signaling dynamics; day-level cell division, apoptosis, and local migration; week-level drug responses; and month-level tumor growth.

The spatial characteristics of the 3D myeloma cell distributions was described by the local cell metrics [33] we previously developed and the corresponding parameters estimated using the 3D cell co-culture system levitated by magnetic field and nano-shuttles [34].

## Results

### Model development

**Figure 1:**
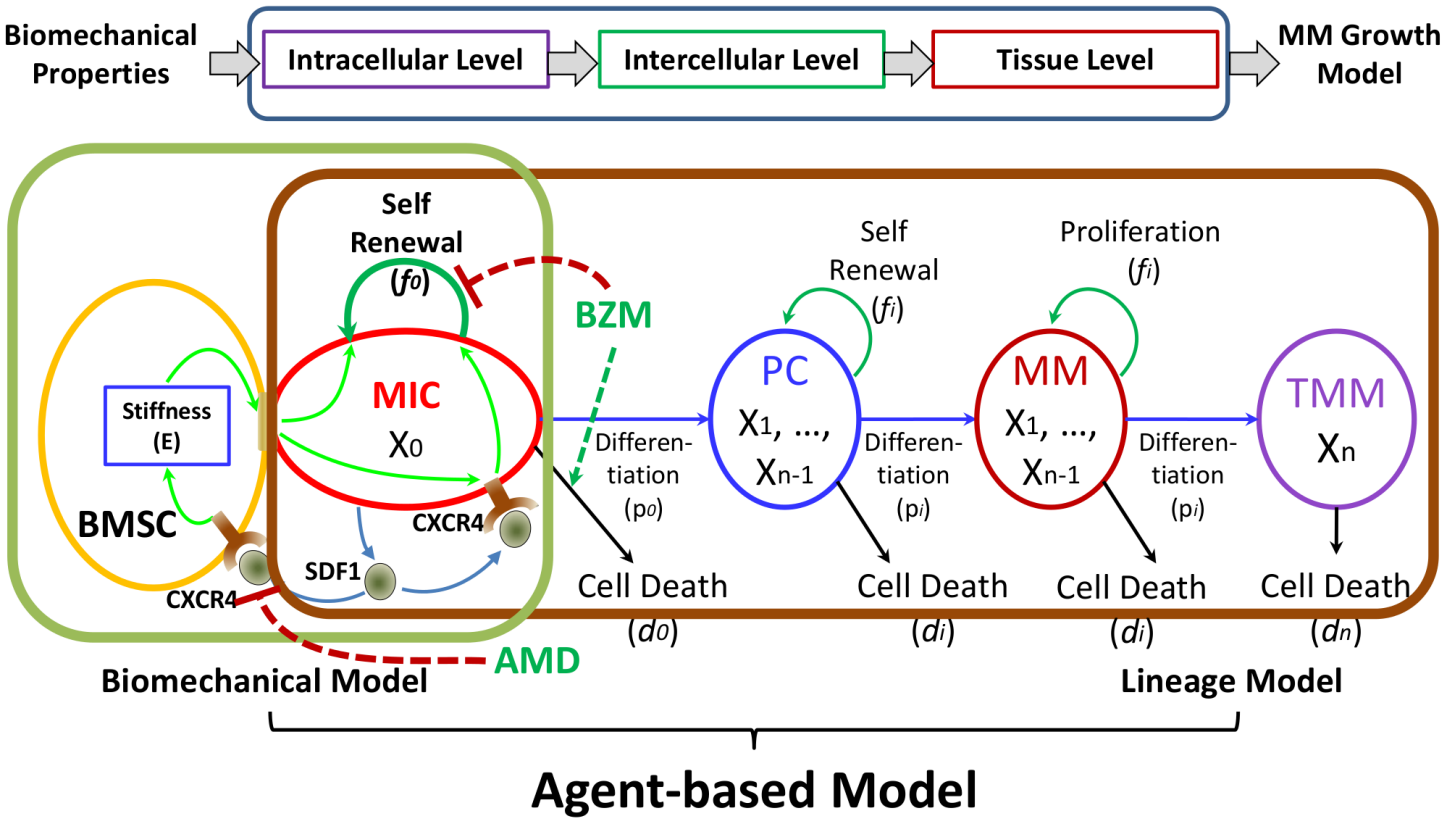
A sketch of ABM model.

We established an agent-based multi-scale model to simulate the development of myeloma in various bone marrow microenvironments in three-dimensional space, and validated the model with experimental data. The *central hypotheses* are: 1) MICs drive the development of multiple myeloma; and 2) the SDF1-centric bio-physical and chemical positive feedback loop boosts MIC growth and colonogenesis, and protects MICs from drug treatments. As shown in **Figure 1**, myeloma development was simulated at intracellular, intercellular, and tissue levels. In this model we included five types of cells which were represented by five types of agents of encapsulated intracellular signaling events and interfaces through which these agents communicated with their microenvironments:

1. Bone marrow stromal cell (BMSC) agent. This agent modeled the 3D reticular network formed by BMSCs. BMSCs changed its biophysical stiffness by cell contraction in response to SDF-1 hints in microenvironment and thus altered the three-dimensional distribution of bone marrow stiffness;
2. Myeloma initiating cell (MIC) agent: This agent represented myeloma stem cells enriched by side population staining [4], [35]. MIC sensed the stiffness of its microenvironment and accordingly modulated its proliferation, differentiation, apoptosis, and drug resistance;
3. Progenitor cell (PC) agent: This agent simulated the expansion of tumor tissue from MICs with given passenger limits. This type of cells was lack of clear surface markers;
4. Mature multiple myeloma cell (MM) agent: The mature multiple myeloma cells which presented clear surface markers (for example CD138) while still proliferating; and
5. Terminal myeloma cell (TMM) agent: modeling terminal MM cells which can no longer proliferate.

Please note that although mathematically PC, MM, and TMM can be lumped into one agent, each of them have unique biological behaviors (such as apoptosis rates) and can be experimentally measured by flow cytometry. Therefore we kept them distinguished for better understanding the simulation results for future experimental validations, and for clinical applications.

At the intercellular level the communication among agents were simulated by the biomechanical model representing the MIC-BMSC positive feedback loops and the myeloma lineage model illustrating the dynamics of different myeloma populations. Interactions between BMSC and MIC were: (a) MICs secreted SDF1 into the neighborhood extracellular matrix. The diffusion of SDF1 in the tissue defined the biochemical microenvironment in bone marrow. The BMSCs contracted according the paracrine SDF1 in their neighborhood and altered the biophysical properties of the bone marrow. MICs sensed and preferably attached to stiffer BMSCs. When attached, stiffer BMSCs boosted proliferation and self-renewal of MICs, and thus promoted the expansion of both MIC and myeloma, and drove sustainable multiple myeloma cancer growth. Stiffer BMSCs also protected MICs from the treatment of cytotoxic drugs.

At the tissue level, the SDF1 profile defined by MIC paracrine and diffusion and the drug concentration defined by the administration of chemotherapeutics determined the biochemical microenvironment in bone marrow, the tissue stiffness defined by BMSC contraction determined the biophysical microenvironment, and myeloma proliferation and migration determined the cellular distribution in bone marrow.

Multiple myeloma cells at different stage of differentiation as defined above were initially seeded at the center of the bone marrow, 100 cells for each cell type, to mimic the initiation stage during myeloma spreading to new locations or after implantation into the bone marrow of animal models. Bortezomib (BZM) and AMD 3100 were used as the representatives of cytotoxic drugs and inhibitors against MIC-BMSC interactions, respectively. After 200 hours of tumor seeding these two drugs were delivered intravenously into the simulated extracellular matrix at combinations of various doses to eliminate multiple myeloma cancer cells. The chemotherapy was scheduled that each cycle included a 400-hour treatment period followed by 500 hours rest (about 2 week treatment followed by 3 week rest), which was typical in clinics. In our experiments usually the animals were sacrificed and the outcome was examined 200 to 400 hours after the first treatment period or one month after tumor xenografting, so the simulation results of the first 600 to 1,000 hours (about one month) were shown. We only simulated the tumor responses to the first treatment cycle because multiple myeloma quickly develops drug resistance and therefore elimination of all MICs in the first treatment cycle is crucial. Parameters were directly determined or indirectly inferred based on our previous experimental results [17], [18], [36], or set according to the best of our knowledge of multiple myeloma, and were summarized in Table S1 and Table S2 in Supporting Information.

To reduce the computational cost the diffusion of the two drugs in bone marrow was assumed to be instantly. This assumption is valid since the myeloma-enriched regions in bone marrow are well vascularized and the diffusion of small molecule drugs is significantly faster comparing with either typical cell behaviors such as migration, proliferation, and apoptosis, or comparing with the diffusion of SDF-1.

Twelve dose levels (including level 0) for each drug and their full combinations were examined under both myeloma-associated BMSC (MBMSC) and normal BMSC (NBMSC) microenvironments, each condition simulated for 20 times. Time resolution (time step) was 2 hours. Totally 288 conditions was explored by 5,760 simulations using parallel computation on a Dell™ PowerEdge™ 850 server of 48 CPU cores.

**Figure 2:**
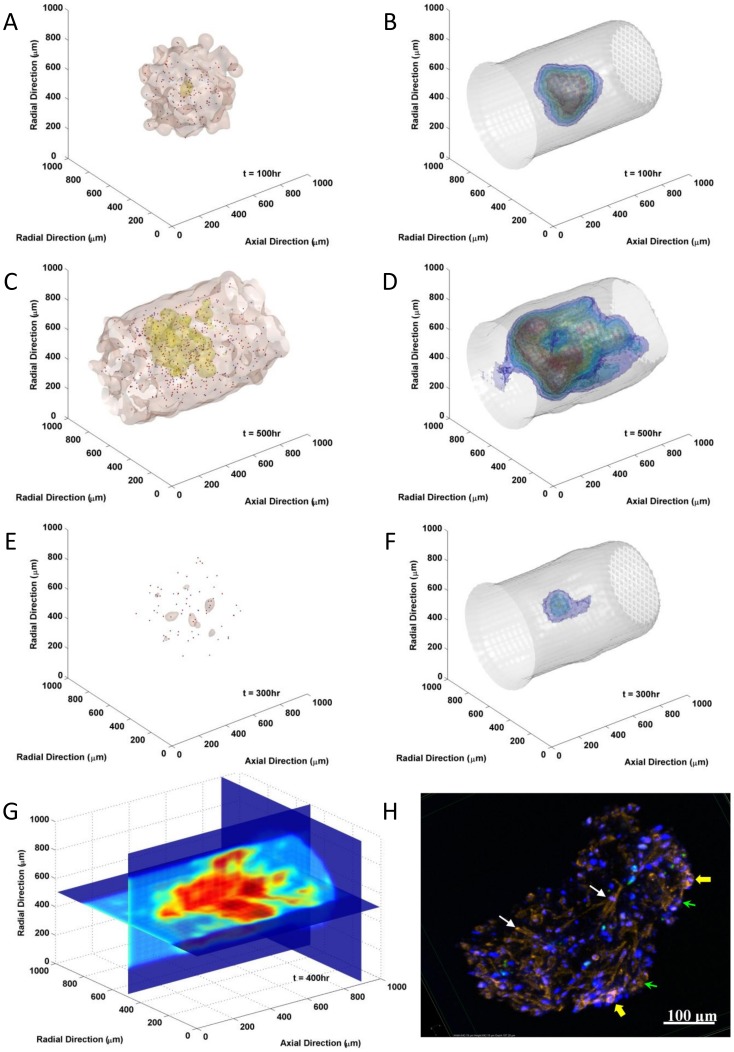
Simulation of myeloma development in three-dimensional bone marrow space. The tumor growth (A and C) associated with the stiffness profiles (B and D) and the activities of MICs at early (100 hr, A and B) and later (500 hr, C and D) stages. (E and F) Tumor and stiffness distributions after BZM treatment at relative level 10 between time point 200 hr and 300 hr. The tumor infiltrating frontiers were labeled by brown isosurfaces, condensed tumor region in yellow, and quiescent MICs in blue while proliferating MICs in red. The stiffness distributions were labeled with isosurfaces, blue denoted lower stiffness and red higher stiffness. (G) The distribution of stiffness in myeloma-associated bone marrow stiffness 400 hr after initial myeloma seeding. (H) The distribution of myeloma cells and the activation of MBMSCs after 4 days 3D levitated culture. Blue: cell nuclei (stained with DAPI); red: F-actin fibers (stained with Alexa Fluor® 594 conjugated phalloidin); white arrow: MBMSCs; green arrow: myeloma cells; yellow arrow: activated BMSCs marked by the formation of stress fiber.

Examples of model simulation results of MIC-driven myeloma growth in bone marrow were visualized and compared with *in vitro* 3D levitated culture image in **Figure 2**. The tumor growth at different stage (100 hr *vs.* 500 hr post-initiating) as well as the associated variations of biophysical properties in bone marrow and activities of MICs were shown in **Figure 2 A** through **D**. As contrast, tumor and stiffness distributions under BZM treatment (dose of the 10^th^ relative level) between 200 and 300 hr were demonstrated in **Figure 2 E** and **F**. Quiescent MICs were highlighted in blue and proliferating MICs in red. The details of biophysical stiffness in bone marrow were visualized by the sections in **Figure 2 G**. Stiffness was labeled in color, from blue to red with respect to the increase of stiffness. Since *in vivo* 3D imaging of BMSC contraction is yet unavailable, we co-cultured myeloma with BMSCs *in vitro* using the 3D levitation system to mimic the bone marrow microenvironment, and 4-day co-cultured tumor tissue was stained and 3D imaged by confocal microscopy (details see **Methods** section) and shown in **Figure 2 H**. BMSCs (white arrows) and myeloma cells (green arrows) were recognized by cell shapes according to F-actin staining (red) as well as nuclei staining (blue). The primed MBMSCs (yellow arrows) were recognized by the formation of stress fibers. The size of the experimentally cultured tumor at day 4 was about 266×492 µm, which was very close to the diameter of the infiltration frontier (about 460 µm) of the simulated results at time point 100 hr (**Figure 2 A**).

Details of the model can be found in the **Methods** section. Typical movies as **Movie S1**, **S2**, and **S3** can be found in **Supporting Information**, and the corresponding simulation conditions in the caption of **Figure 2**.

### Model validation

**Figure 3:**
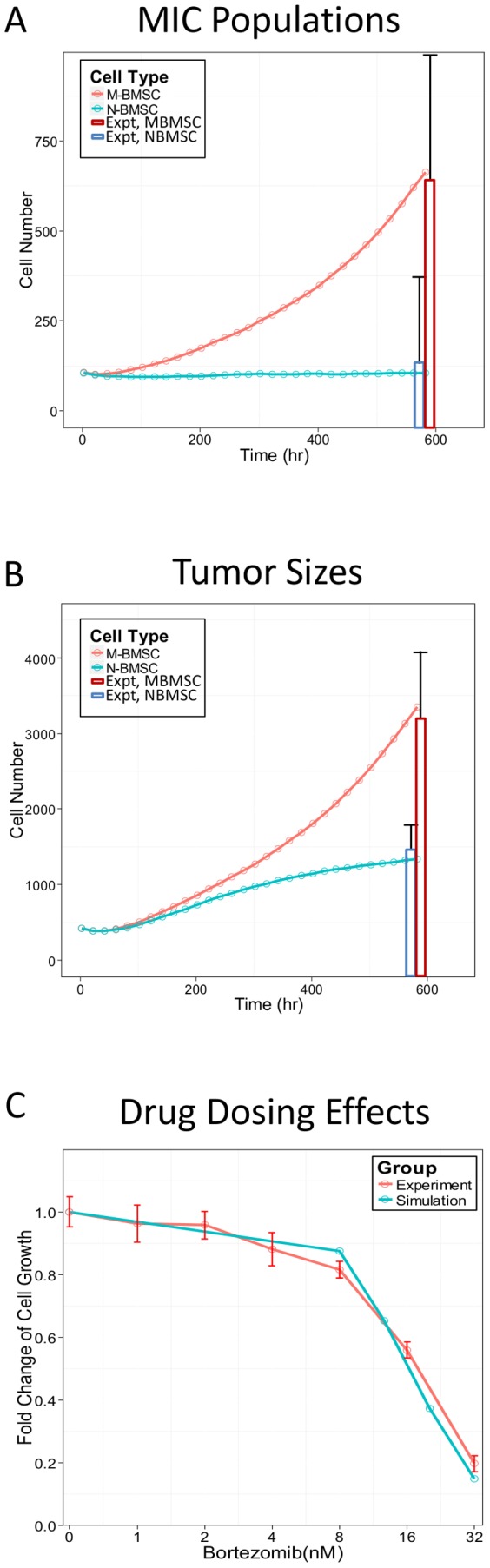
Comparison of simulation results and experiment observations. The simulation results of the MIC populations (A) [18] and the total tumor sizes (B) [18] under NBMSC or MBMSC environments as well as the dose responses in terms of total tumor size decrease (C) [36] were compared with our published experiment results.

The simulation results of the agent-based model of myeloma growth under different biophysical microenvironments were compared with experimental results [18], [36] to validate the model. The simulated MIC populations as well as the total tumor sizes under the NMBSC and MBMSC (Blue *vs.* red lines in **Figure 3 A** and **B**) after 4 weeks growth were consistent with experimental results [18] (Blue *vs.* red bars in **Figure 3 A** and **B**). Tumor also showed consistent BZM dose responses after 48 hr treatment in simulation and in experiments (blue *vs.* red lines, **Figure 3 C**). Data shown in **Figure 3** were the mean values of the 20 simulations for every 20 hours (every 10 time steps).

### Effects of MBMSCs in disease development

**Figure 4:**
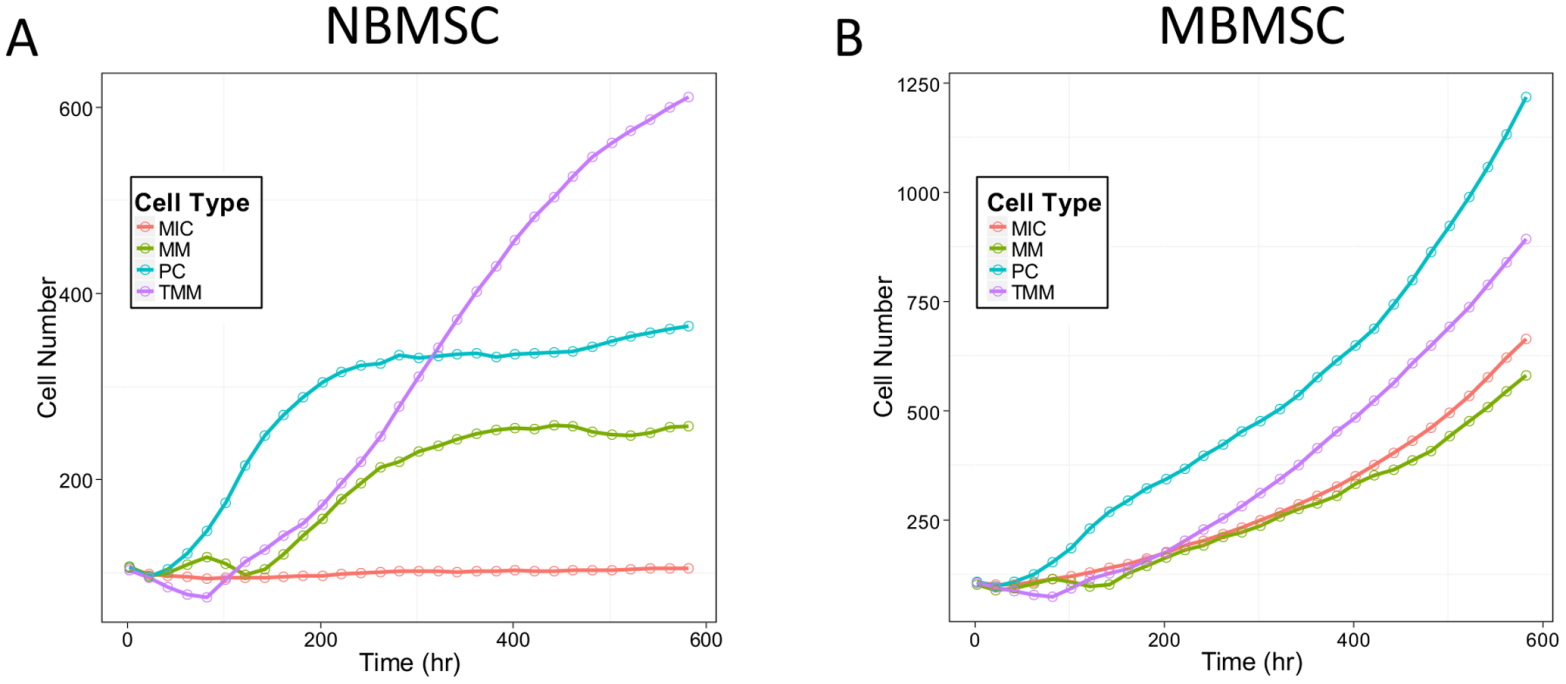
Profiles of simulation results. The growing trends of each tumor cell type under (A) NBMSC or (B) MBMSC environments were visualized.

We first tuned the model to recapture experimental observations and explored the roles of myeloma-associated MBMSCs in disease development using the NBMSCs as controls. The simulation results of tumor growth and the comparison with experimental data were summarized in **Figure 4** and **Figure 3**. The cell number of each tumor cell type during the first 600 hours (25 days) were shown for the NBMSC (**Figure 4 A**) or the MBMSC (**Figure 4 B**) case, and the MIC populations (**Figure 3 A**) as well as the total tumor sizes (**Figure 3 B**) were compared with our previously published experimental results. Tumor grew about 2.6 folds faster under MBMSC context, while the MIC population was 6.8 folds larger. Simulation results were consistent with experiments. The myeloma population in MBMSC was dominated by “younger” tumor cells such as progenitors comparing with the normal counterpart, which was dominated by terminal myeloma cells. Data shown in **Figure 4** were also the mean values of the 20 simulations for every 20 hours (every 10 time steps).

### Effects of MBMSCs on chemotherapy outcomes using BZM

**Figure 5:**
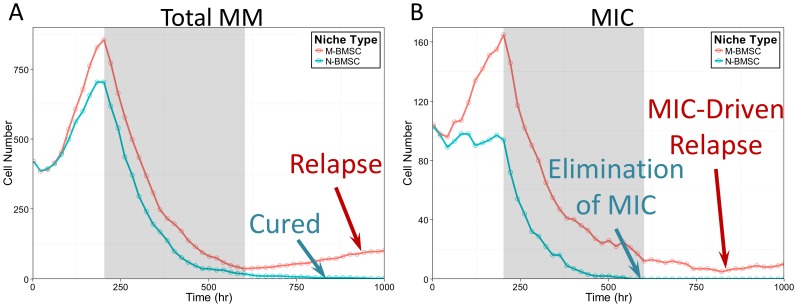
MIC-driven myeloma relapse after cytotoxic chemotherapy cycle. (A) The growing trends of tumor cells under MBMSC (red line) *vs.* NBMSC (blue) environments under drug treatments. (B) The growing trends of MICs under MBMSC (red line) *vs.* NBMSC (blue lines) environments under drug treatments. Drug treatment periods were highlighted with gray shade.

To explore the drug resistance to chemotherapies and the risk of tumor relapse in MBMSC and NBMSC microenvironments, we simulated the first BZM treatment cycle at 11 dose levels covering two magnitudes. BZM was delivered 200 hours after tumor initiation and the treatments lasted for 400 hours. The dynamic of total tumor cells as well as MICs during the first 1000 hours were shown in **Figure 5**. After drug delivery, the tumor populations quickly dropped for both MBMSC and NBMSC (red and blue lines in **Figure 5 a**, respectively) cases. The drug efficacy, in terms of the elimination of myeloma population, was similar in both cases, and during the first treatment cycle the tumor cell number decreased to a very low level that were clinically not detectable. However, drug efficacy on MICs, a small portion of the tumor population, was significantly different. Medium dose of BZM treatment (the sixth level among all eleven) completely killed all the MICs in NBMSC-dominated bone marrow (**Figure 5 b** blue line), whereas a few MIC cells survived the chemotherapy in the MBMSC case (**Figure 5 b** red line) due to the drug resistance boosted by myeloma-associated stroma. The MIC-free tumor population kept degenerating till the cure of the disease, while in contrast the survived MICs re-initiate the myeloma and caused MIC-driven tumor relapse (**Figure 5 a** blue *vs.* red lines, time 600 to 1000 hr).

Two typical simulations were shown in **Figure 5**, and the rest were discussed in the following sections.

### Inhibition of MIC-BMSC communications by AMD 3100 treatment

**Figure 6:**
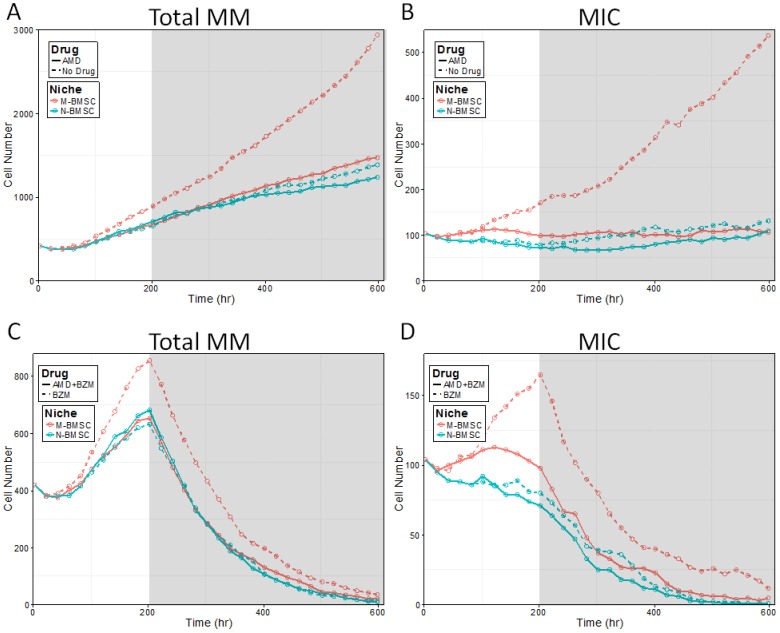
The effects of MIC-BMSC interaction inhibition on tumor growth and the outputs of cytotoxic chemotherapy. (A) The growing trends of tumor cells under MBMSC or NBMSC environment with or without niche inhibitor (AMD3100). (B) The growing trends of MIC cells under MBMSC or NBMSC environment with or without niche inhibitor (AMD3100). (C) The responses of myeloma tumor to cytotoxic drugs under MBMSC or NBMSC environment with or without niche inhibitor (AMD3100). (D) The responses of MICs to cytotoxic drugs under MBMSC or NBMSC environment with or without niche inhibitor (AMD3100). Myeloma-associated or normal BMSC niches were denoted in red or green color, and the effects of the niche inhibitors on tumor growth (A and B) or cytotoxic drug efficacy (C and D) were labeled in solid lines and their controls in dashed lines, respectively.

**Figure 7:**
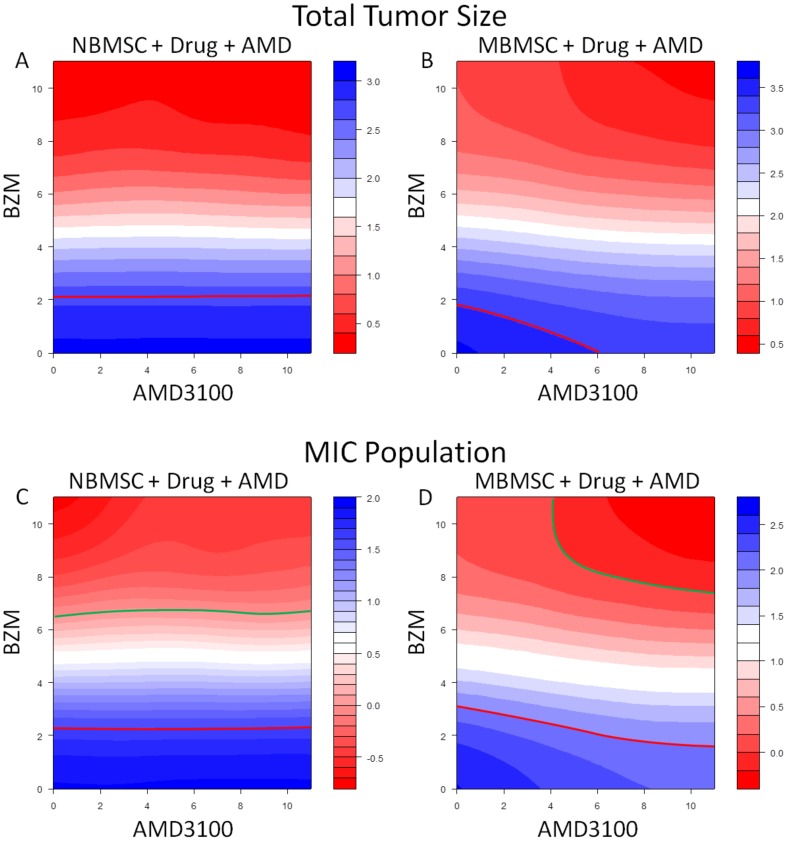
Synergy Effects of AMD3100 and BZM on total myeloma size (A and B) or on MICs (C and D) in NBMSC (A and C) or MBMSC niches (B and D). Red lines indicate the E50 isoboles and green lines mark the E100 isoboles used in Loewe drug combination analysis.

We then examined if the inhibition of MIC-BMSC communications could deprive MICs from the protection of their stroma and re-sensitize the tumor in the MBMSC context to chemotherapy. AMD 3100, a competitive inhibitor of SDF-1, was delivered at 11 levels crossing two magnitudes, either alone or with BZM, to myeloma growing with either myeloma-associated or normal BMSC stroma. The effects of medium AMD dose were visualized by two simulations in **Figure 6** and the rest summarized in **Figure 7**. AMD-treated and AMD-free cases were denoted in solid and dashed lines, and the myeloma-associated and normal BMSC niches in red and blue colors, respectively. Consistent with **Figure 3 (A)** and **(B)** and **Figure 5 (A)** and **(B)**, MBMSC niches promoted tumor growth (**Figure 6 a** red *vs.* blue dashed lines), MIC self-renewal (**Figure 6 b** red *vs.* blue dashed lines), and drug resistance (**Figure 6 c** red *vs.* blue dashed lines) driven by MICs (**Figure 6 d** red *vs.* blue dashed lines). Introduction of niche inhibitor at moderate level alleviated all these effects (**Figure 6 A** through **D** red *vs.* blue solid lines).

### Combined drug effects on both cases

To evaluate if interrupting the MIC-BMSC communications has clinical potentials for multiple myeloma, we tested the combinatorial effects of the two representative drugs, AMD3100 and BZM. For each drug, 12 doses (0 as control and 11 levels from 0.1× to 10× in geometric sequence relative to the original dose) were selected and the full combinations of the two drugs were explored for drug efficacies. The default doses for each, denoted as 1×, was defined as the minimum doses that, for BZM, eliminated all MICs in NBMSC case and, for AMD3100, deprived MBMSC support. Each combinatorial condition was simulated for 20 times at 400 time points for 5 cell types and SDF-1 concentration distributions, and totally 13,824,000 data sets were generated. The means of replicates were used for further analysis, and the synergistic effect maps were further smoothed with a two dimensional Gaussian kernel (θ = 2) for robust visualization.

Loewe combination index [37], [38] (for an introduction and a guide of interpretation of Loewe combination index please refer to **Figure S1** in the **Supporting Information**), defined according to the 50% MIC population reduction by the end of the two week chemotherapy (E50 at time point 600 hr), was utilized to evaluate the synergism of AMD3100 and BZM while treating myeloma in either NBMSC or MBMSC niche. We selected the total myeloma death rate as well as MIC death rate as the indicator of drug efficacy because as demonstrated in **Figure 5** (first and second rows, respectively). MIC played essential role in post-treatment disease relapse [4]. The SDF-1/CXCR4 inhibitor showed on moderate effects on the efficacy of BZM when treating multiple myeloma developed in both normal and myeloma-associated bone marrow stromal cell microenvironments in terms of both the shrinkage of total tumor size (red contour lines in **Figure 7 a** and **b**, respectively) and the decrease of MIC populations (red contour lines in **Figure 7 c** and **d**, respectively). In contrast, dramatic synergistic effects of the two drugs were observed if the total elimination of MIC (E100) was used as the criterion for Loewe combination index in MBMSC cases (green contour lines in **Figure 7 d**) but not in NBMSC cases (green contour lines in **Figure 7 c**).

## Discussion

This work previewed the druggability of the biophysical features of cancer stem cell niches, and cast new light on the strategies to overcome the drug resistance and relapse of multiple myeloma.

The central hypothesis of this work, that the positive feedback loop between MIC and MBMSC *via* SDF-1 paracrine and the increase of MBMSC niche stiffness promotes myeloma development and is responsible for drug resistance and cancer relapse, has been successfully realized in this multi-scale agent-based model. **Figure 2** showed that MBMSCs close to MICs were primed and activated by SDF-1 secreted by MICs provided a stiffer microenvironment; such pro-ontogenetic niches in turn boosted MICs in terms of proliferation and drug resistance.

It was the first time the dynamics of the bone marrow stiffness during the development of myeloma was simulated and visualized in three-dimensional space at multiple scales. While the *in vivo* assay technologies of the bone marrow biophysical properties are yet not available, this model provides insights into the mutual communications between cellular and mechanical information. The simulations (**Figure 2** and **Supporting Information Movie S1** and **S2**) suggested that both the infiltration process of myeloma as well as the distribution of bone marrow stiffness were highly dynamic and the two types of distributions showed temporal and spatial lags. *In vivo* monitoring the changes of bone marrow biophysical properties can be a valuable method to directly estimate drug efficacy of myeloma-associated BMSC inhibitors.

Our simulation results also emphasized the crucial role of MICs in disease relapse, which was illustrated in **Figure 5**. The elimination of cancer stem cell is the essential goal of chemotherapies for multiple myeloma; otherwise the survived MICs will drive the re-initiation of the cancer and the relapse of the disease [4]. However, due to the drug resistance of MICs and the protection of MIC niches, it is challenge for traditional chemotherapies to target and purge all MICs [4], [39]. Targeting the MIC niches thus becomes an intriguing strategy.

Besides that the model is conceptually consistent with our knowledge of multiple myeloma, we also confirmed that the simulations reproduced the key experimental findings of MIC/MBMSC interactions [17], [18], [36]. Although parameters of this piloting model were roughly and bona fide determined from literatures, our experiments, and the best of our biological knowledge (**Table S1** and **Table S2** in **Supporting Information**), the model is capable to re-capture our experimental observations (**Figure 2 H** and **Figure 3**).

1. Tumor initiation. One major feature of multiple myeloma is its frequent metastasis. Understanding the initiation and early stage development of myeloma at new bone marrow sites is thus critical for clinical intervention. However, it is yet challenging to monitor such events *in vivo*. We established the 3D levitated co-culture system to mimic the early events of the development of secondary myeloma and to reveal its unique features. The similar growth trends from the simulated results and the 3D co-culture in terms of tumor size (**Figure 2 A** *vs.* **H**) suggested that the model is capable of predicting growth trends after successful metastasis as well as the post-treatment re-initiation of tumors. The geometric shapes of myeloma growth are dynamic and subject to the stochastic feature of cell migration as well as the context of cell growth, though.
2. Pro-oncogenetic MBMSC microenvironments. As mentioned before, accumulating evident suggest that MICs were capable to prime MBMSCs but not NBMSCs in terms of BMSC stiffness, which in turn promotes myeloma growth and enhance drug resistance. The model re-captured these features. MICs population was boosted in myeloma-associated bone marrow (red lines in **Figure 4 B** versus **A** and the red *vs.* blue line in **Figure 3 A**) and thus drove the development of myeloma (**Figure 3 A** versus **Figure 4 A** and the red *vs.* blue line in **Figure 3 B**).
3. Drug responses. The consistent dosing effects of BZM in terms of the shrinkage of tumor sizes between the model predictions and the experimental results (**Figure 3 C**) are strong evidence that the model has the capability to predict the drug responses of myeloma over a wide range of drug doses. AMD3100 deprived the support of niches to the expansion of MICs (**Figure 6** solid *vs.* dashed lines). MICs showed drug resistance comparing with other myeloma cells [4], [39], and such drug resistance was enhanced at the presence of MBMSCs (**Figure 5** red *vs.* blue lines) [18].

The simulation results were consistent with our and others experimental observations, which provided a solid basis for the exploration of the synergistic effects of niche-specific drugs with cytotoxic chemotherapeutics. Please notice that although the *in vitro* 3D levitated culture has been known to be able to mimic *in vivo* microenvironment [34], the bone marrow contexts are still significantly different from the *in vitro* environments. Therefore, the highly consistent results between experimental data and simulation (**Figure 2 A** *vs.* **H**) only suggested that the simulation results were reasonable and the model is sufficient for piloting *in silico* study. *In vivo* assays of myeloma growth and drug responses are still necessary for parameter estimation, model training, and accurate predictions, which is future work and is beyond the focus of this study.

Loewe drug combination analysis [40], [41] strongly suggested that inhibitors that block the MIC/BMSC communications are of high clinical potentials. Traditional Loewe combination analyses for chemotherapeutics often focus on the bulk tumor and use isobole at effect level 50 (E50) as standard index, which is convenient to monitor in animal models and clinical chemotherapy evaluations using micro-CT, bioluminescence, biopsy, and others. The elimination of the bulk tumor is also used as clinical index. However, this standard may not accurately reflect the drug efficacies against tumor-initiating-cell-driven tumor development. Capability to eliminate of all tumor initiating cells should be the key evaluation index. We thus defined the E100 isobole against MICs in our Loewe analysis and explored drug combinations that could successfully kill all MICs in the first treatment cycle. Simulations of the combination effects of the cytotoxic drug (BZM) and the niche-specific inhibitor (AMD3100) in myeloma-associated stroma, evaluated by the E100 isoboles against MICs, suggested strong synergistic effects (green lines in **Figure 7 D**). In contrast, E50 isoboles only indicated slight synergism between the two drugs (red lines in **Figure 7 A** through **D**). The control groups of the normal BMSC environments showed no responses to niche inhibitors (**Figure 7 A** and **C**), which further confirmed that AMD3100 did not directly affect MICs and the strong synergism observed in the MBMSC cases were due to the interruption of the MIC-BMSC communication. Such dramatic difference suggested that for TIC-driven tumors drug effects should be estimated in terms of the total eradication of TICs, and E100 isoboles against TICs provide a more sensitive evaluation of the effectiveness of treatments. Specifically, for the multiple myeloma treatment evaluation and relapse risk estimation, our results highly suggested using the percentage of MIC population in patient bone marrow aspirate by either side population staining or surface marker immuno-staining (e.g. CD138) and flow cytometry.

Simulations also suggested that niche-specific drugs are strongly synergistic with tumor-specific cytotoxic drugs and re-sensitize myeloma to the otherwise resistant drugs. Cytotoxic drugs alone, though may efficiently kill the bulk of tumor, sometimes are not sufficient to demolish all TICs. As shown in **Figure 7 D**, without the aid of AMD3100, BZM alone cannot extinguish all MICs and thus the disease will relapse soon after the chemotherapy (**Figure 5**). Taken together, the physical properties of the myeloma initiating cell niches is highly targetable, and the inhibitors of the interactions between cancer initiating cells and their stroma are promising co-drugs for traditional cytotoxic agents.

As a piloting research, this computational model is conceptual and some parameters were not directly estimated from experiment data, and some in vivo/clinical data and assays such as drug delivery and metabolism in patients may improve the accuracy of the predictions. However, we have omitted such details due to the following reasons: 1) To keep the focus of this work of the preliminarily modeling of a novel system. It is the first time to simulate the druggability of the biophysical properties, mainly the stiffness, of the bone marrow microenvironments and the major focus of this work is to establish a reliable model according to the relatively solid in vitro and animal experiment data. At this preliminary stage, it may be not ready to reach a precise predictive model for clinical treatment outcomes by including clinical data about the bone marrow properties as well as the underlying mechanisms (such as the diffusion processes of cytokines and the pharmacokinetics and the pharmacodynamics of bortezomib and AMD3100). 2) To address our major questions and reach conclusions with a reasonably simple model as possible. We have also simplified the detailed temporal profiles of drugs during chemotherapies and adjuvant therapies to constant levels as such detailed dynamic features, though important for providing optimal dosing in clinical treatments in the future, are not our focus at this stage. Indeed such simplification approaches are common in preliminary models of new systems and do not change the main conclusions of this work. 3) Furthermore, detailed molecular mechanisms of underlying signaling pathways are still missing before we can reach an accurate and more predictive model. The major goal of this work is to extend our current knowledge of how MICs modulate the physical properties of their niches to evaluating the therapeutic potential of targeting such remodeling process. For such purpose, the results of this work suggest using the complete eradication of TICs as drug efficacy index for TIC-driven cancers, emphasize the importance of targeting TIC niches, and encourage following-up investigations of niche-specific therapeutics.

### Conclusions

It was the first time the three dimensional modeling of the biophysical properties of cancer stem cell niches and the therapeutic potential of targeting such mechanical features were attempted at the system level. Our *in silico* model successfully realized the bio-model of the MIC-BMSC chemical-physical positive feedback loop, and was capable to capture the key features of our experimental observations. Simulations suggest that the isoboles of drug capability to completely eliminate TICs in Loewe combination analysis can better predict treatment outcomes. Drug synergism analysis suggested that intervening the communications between myeloma initiating cells and their niches dramatically enhanced the efficacy of cytotoxic drugs against myeloma initiating cells, re-sensitized multiple myeloma to chemotherapies, and reduced risks of cancer relapse.

## Methods

**Figure 8:**
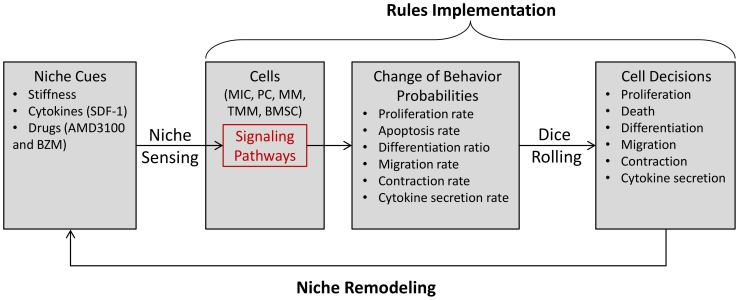
The stochastic simulation of cell behaviors using rule implementation, dice-rolling-based decision-making, and Markov chain Monte Carlo approaches.

We defined five types of agents in the model to represent BMSC, MIC, PC, MM and TMM. We initialized the bone marrow as a cylinder 3D rectangular framework, with the tumor extracellular matrix (ECM) filled with BMSC agents distributed evenly across the 3D ECM and the mixed MIC, PC, MM and TMM agents at the center of the bone marrow as a sphere. Such initiating cell ratios were according to the myeloma initiating cell hypothesis as well as our previous publications that the MICs can restore the whole myeloma population, including the composition ratios of myeloma cell types. This multi-scale modeling consisted of three scales: intracellular, intercellular and tissue scales, which were illustrated in **Figure 1**, **Figure 8**, and described in details in the following sections. The model was generally presented as pseudo-code and listed in **Figure S6**. Detailed flowcharts of each myeloma agents were illustrated in **Figure S2** (for MIC agent), **Figure S3** (for PC agent), **Figure S4** (for MM agent), and **Figure S5** (for TMM agent) in the **Supporting Information**. Parameters were determined according to our previous work as well as literature [17], [18], [36]. Mathematical and computational details are elaborated in **Support Material**.

### Stochastic Simulation of Cell Behaviors

The Markov Chain Monte Carlo approach was used to simulate cell behaviors of each individual cell. As shown in **Figure 8**, cell behaviors were simulated by probability-based rule implementation. A cell sensed the hints of its neighborhood such as stiffness, SDF-1 level, and drug doses, processed them with imbedded pathways, and outputted the changes of probabilities of corresponding cell behaviors including cell proliferation rate, apoptosis rate, differentiation ratio, migration rate, contraction rate, and cytokine secretion rate. Cell decision was then determined by rolling a dice and compared with the probability of a given cell behavior. Cell behaviors in turn remodeled the properties of its niches. Details of each cell behavior for each type of cell agent as well as the corresponding rule was discussed in the following sections as well as the **Supporting Information**.

### Intracellular Scale

Each BMSC agent encapsulated signaling pathway functions to determine its biomechanical phenotype switch, using an agent-specific Hill function [42] to describe the SDF-1/CXCR4 signaling pathway which regulated the local stiffness in response to the in-situ relative SDF1 concentration. The effects of the SDF1/CXCR4 inhibitor AMD3100 was simulated by competing with SDF1 for CXCR4 and thus reducing the effective SDF1 concentration.

Meanwhile, the responses of tumor agents (MIC, PC, MM, and TMM) to local ECM stiffness in terms of the possibilities of cells to enter the proliferation, apoptosis, and migration status were also simulated using Hill functions in similar ways. Cell decision-making process was defined by agent rules with such probabilities as the major inputs. The stochastic feature of the decision of an individual cell was realized by die casting simulation.

#### Stem cell fate determination

Once a MIC decided to enter cell cycle, its fates were further determined according to its micronenvironment. A MIC either generate two MIC daughter cells, known as self-renewal, or to two PC cells, know as differentiation, or to one MIC and one PC, known as asymmetric division. The probability of each fate was determined using Hill functions, and the decision of each MIC agent was also realized by die casting simulation as mentioned above.

#### Proliferation fates of PC and MM agents

The fates of intermediate cell agents were determined by their passage ages as well as the probabilities of proliferation which represent the effects of cell neighborhood characters such as stiffness and cytokine concentration, and the current cell cycle status of the cells. PC agents were different from MM agents for their limited self-renewal capability. When maximum renewal limit reached, a PC agent differentiated to an MM agent. TMM agents did not proliferate. According to the myeloma initiating cell hypothesis, only MICs can self-renew and proliferate without limits, so defined is the LGN, which is the maximum passage number a PC cell can self-renew, or a MM cell can proliferate.

### Intercellular Scale

Once a myeloma cell (MIC, PC, MM, or TMM) had determined its responses to the biomechanical properties of its microenviroment, it would proliferate, migrate, being quiescent, or undergo death, which were described in the intercellular scale.

#### Migration

A non-M-phase cell would migrate if it could find free space nearby. The migration was governed by rules representing space availability, stroma preference, migration speed, and stochastic effects using Hill functions and die-casting simulation as mentioned above.

#### Division of MIC, PC and MM agents

If M-phase cell found at least one free location within searching distance, it would divide, following the same migration rules but with a much smaller migration distance (i.e., slower migration speed) so the de novo daughter cells were always next to the parental cells. If no space was available, cells would remain in M-phase and try in next round.

#### Apoptosis

The decision-making of cell apoptosis was simulated using a pre-defined threshold for the apoptosis rate reflecting cell microenvironment, especially the local drug (BZM and AMD) concentrations and stiffness. The whole apoptosis process took about 20 hours. RPMI 8226 myeloma cell apoptosis rates under drug treatments were determined according to our previous study [36].

### Tissue scales

In the tissue scale of this ABM, the secretion of SDF1 from MIC agents and the diffusion of SDF1 in the 3D ECM defined the dynamic 3D distribution of SDF1 concentration [43]–[45]. SDF1 was uniformly initialized at the start with Dirichlet boundary.

In summary, intracellular signaling pathways were encapsulated into each cell to determine either the BMSC intercellular biomechanical phenotype (cell stiffness) or tumor cells' (MIC, PC and MM) behaviors (migration, differentiation, proliferation, or apoptosis). Cancer cells competed for the best location in 3D extracellular matrix to migrate or proliferate regarding to the change of BMSC cell's stiffness and cell density. In turn, chemo-attractant cues (SDF1) at the tissue level triggered BMSC cell's intracellular signaling pathways by receptors. And the resultant feedbacks were the changes of either cancer cells' properties (change of BMSC cells' stiffness) or behaviors of cancer cells (secretion of cytokines, proliferation, differentiation, apoptosis, or migration). Thus, the 3D dynamics of bone marrow stiffness and tumor growth were simulated at multiple temporal and spatial scales.

## Materials and Methods

### Myeloma cell model

We chose RPMI 8226, one of the most widely used human myeloma cell lines, as the myeloma cell model because it had been shown representative of key myeloma pathogenesis features of interest in this work. *In vivo* and *in vitro* evidence from our [17], [18], [36] and other research groups [3], [4], [46] suggested the clinical translational values [46] of the MICs from RPMI 8226, which re-capture major clinical myeloma stem cell characters such as drug resistance and initiating myeloma in bone marrow by generating the whole myeloma population, both of which are boosted by the positive feedback loops with their associated stroma cells *via* the SDF-1/stiffness chemo-physical interactions. Specifically, RPMI 8226 has been shown to be representative among other frequently used human myeloma cell lines in terms of Bortezomib treatment responses [47], [48].

### Cell isolation and culture

Myeloma-associated and normal stroma cells were isolated and expanded from myeloma patients similarly as previously described [18], depending if they were diagnosed of bone marrow involved myeloma or not. Briefly, after lysis of red blood cells (RBC Lysis Solution, QIAGEN), patient bone marrow aspirate was cultured in MesenPRO RS™ medium (Invitrogen) in 5% CO_2_ humidified atmosphere at 37°C for one week and unattached cells discarded. Attached MBMSC was expended under same culture conditions. Usage of these samples has been approved by the Institutional Review Board of The Methodist Hospital Research Institute (TMHRI). RPMI8226 multiple myeloma cell line was purchased from ATCC and cultured in RPMI 1640 medium (Mediatech) supplemented with 8% fetal bovine serum (Invitrogen) 100 units/ml penicillinand 100 mg/ml streptomycin (Life Technologies) at 37°C in 5% CO2. This study was granted for Consent waiver by IRB.

### Levitated 3D co-culture

Both MBMSC and RPMI8226 were treated with Nanoshuttle™-PL (n3D Biosciences) overnight per manufacturer's instruction, MBMSC detached by slight trypsin treatment, mixed at 1∶20 to 1∶200 ratios (MBMSC:RPMI8226), transferred to Costar® 6 well Ultra Low Attachment plate (Corning) to reach about 400 cells per well, and immediately levitated using the 6-well Bio-Assembler™ Magnetic Drive (n3D Biosciences) and cultured in RPMI 1640 medium (Mediatech) as described before.

### Staining and imaging

At day 4 the assembled MIC/MBMSC co-cultured tissue was collected, rinsed in PBS, fixed with 3.7% paraformaldehyde (Fisher) for 10 min, blocked with 1% bovine serum albumin (Fisher), and stained with Alexa Fluor® 594 phalloidin (Invitrogen) using 1∶40 dilution for 30 min, followed by counter staining using 300 nM DAPI (Invitrogen) for 5 min, rinsed with DI water, and mounted with ProLong® Gold Antifade Reagent (Invitrogen) for imaging. The process was under the protection of the Magnetic Drive to avoid lost of cells. The Nikon A1 Confocal Imaging System was used to image samples for DAPI and Alexa Fluor® 594 signals with optical slicing distance of 8 µM and the 3D images were reconstructed using NIS-Elements Microscope Imaging Software (Nikon).

## Supporting Information

Figure S1 **The Loewe drug combination analysis.**
(TIF)

Figure S2 **The flowchart of the MIC agent.**
(TIF)

Figure S3 **The flowchart of the PC agent.**
(TIF)

Figure S4 **The flowchart of the MM agent.**
(TIF)

Figure S5 **The flowchart of the TMM agent.**
(TIF)

Figure S6 **The pseudocode of the multi-scale agent-based model.**
(TIFF)

Movie S1 **Simulation of myeloma growth in three-dimensional bone marrow space.** Simulation conditions and details see Figure 2.
(MP4)

Movie S2 **Simulation of stiffness profiles associated with myeloma growth in three-dimensional bone marrow space.** Simulation conditions and details see Figure 2.
(MP4)

Movie S3 **Spatial stiffness profile in three dimensional bone marrow space 400 hr after initiation.** Simulation conditions and details see Figure 2.
(ZIP)
