# Correction: Targeting the Biophysical Properties of the Myeloma Initiating Cell Niches: A Pharmaceutical Synergism Analysis Using Multi-Scale Agent-Based Modeling

**DOI:** 10.1371/journal.pone.0112421

**Published:** 2014-10-28

**Authors:** The PLOS ONE Staff

## Notice of Republication

This article was republished on October 17, 2014 to include the supporting information files, which were omitted in the original version. Please download this article again to view the correct version. The originally published, uncorrected article and the republished, corrected article are provided here for reference.

## Supporting Information

File S1 Originally published, uncorrected article.
(PDF)

File S2 Republished corrected article.
(PDF)
