# Supplementary figures and images for: Targeting the Biophysical Properties of the Myeloma Initiating Cell Niches: A Pharmaceutical Synergism Analysis Using Multi-Scale Agent-Based Modeling
Source: PLoS One. 2014 Jan 27;9(1):e85059. doi: 10.1371/journal.pone.0085059 (PMC3903473; doi:10.1371/journal.pone.0085059)

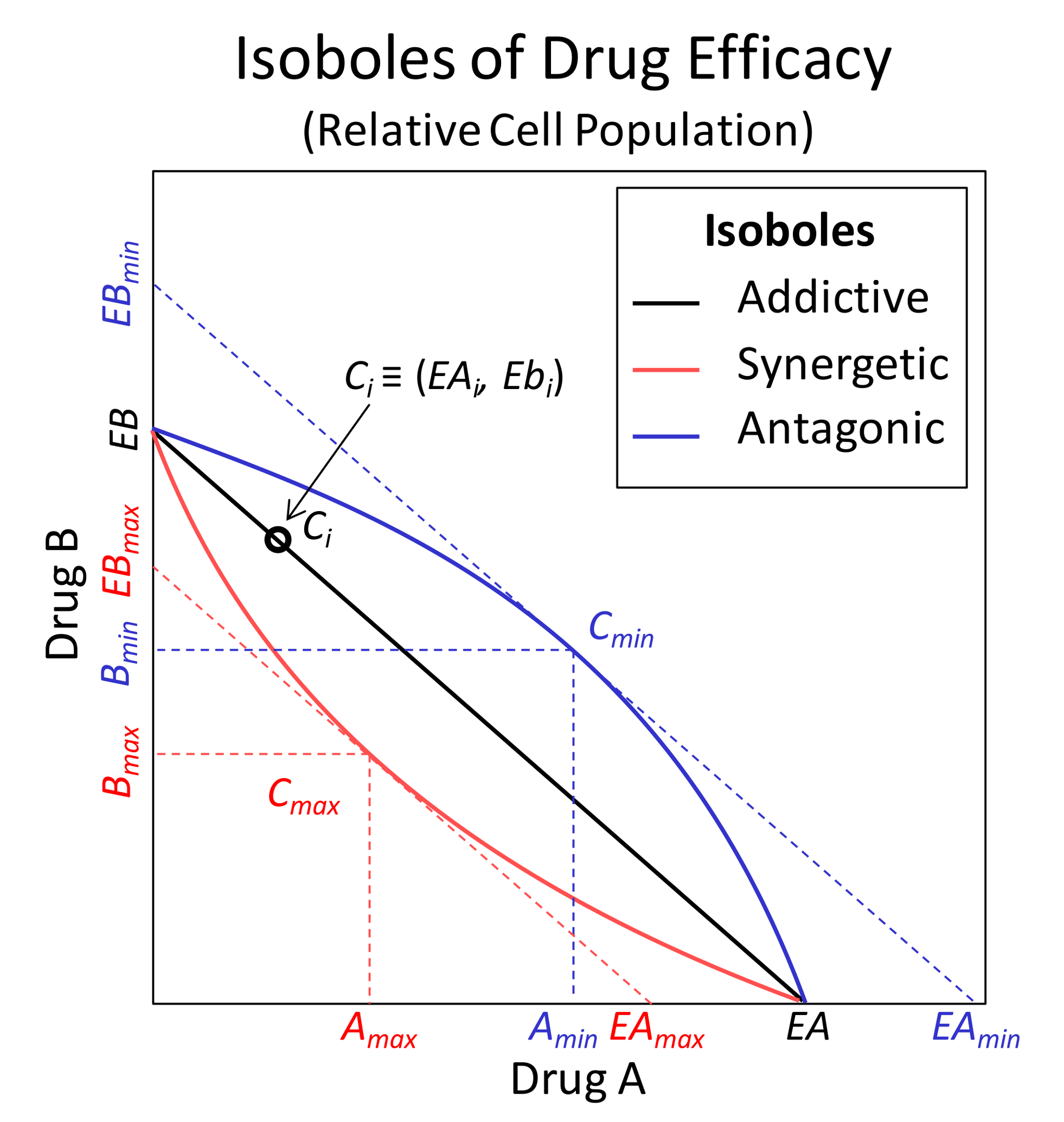

Supplement: Figure S1 — The Loewe drug combination analysis. (TIF) [file pone.0085059.s001.tif]

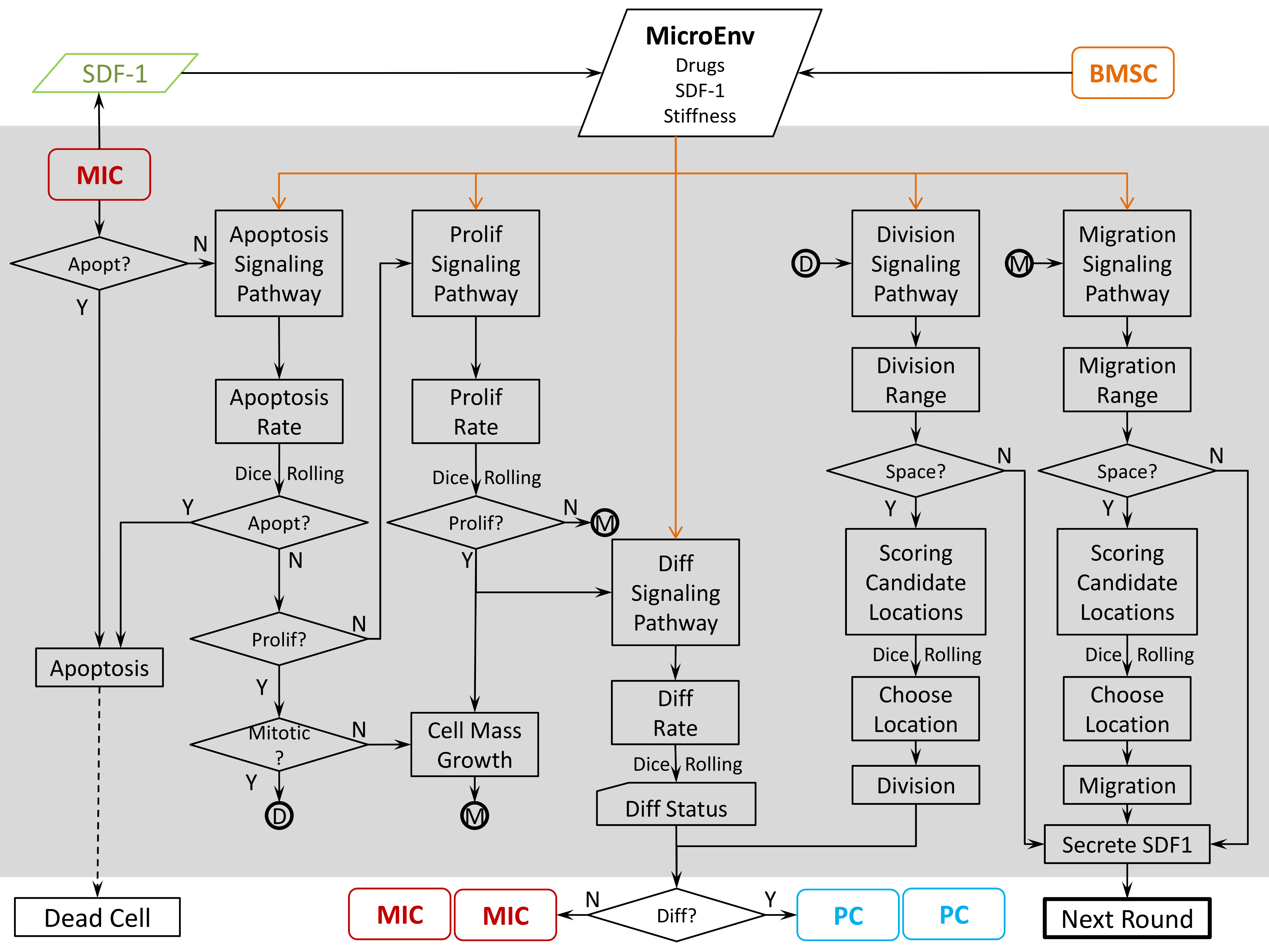

Supplement: Figure S2 — The flowchart of the MIC agent. (TIF) [file pone.0085059.s002.tif]

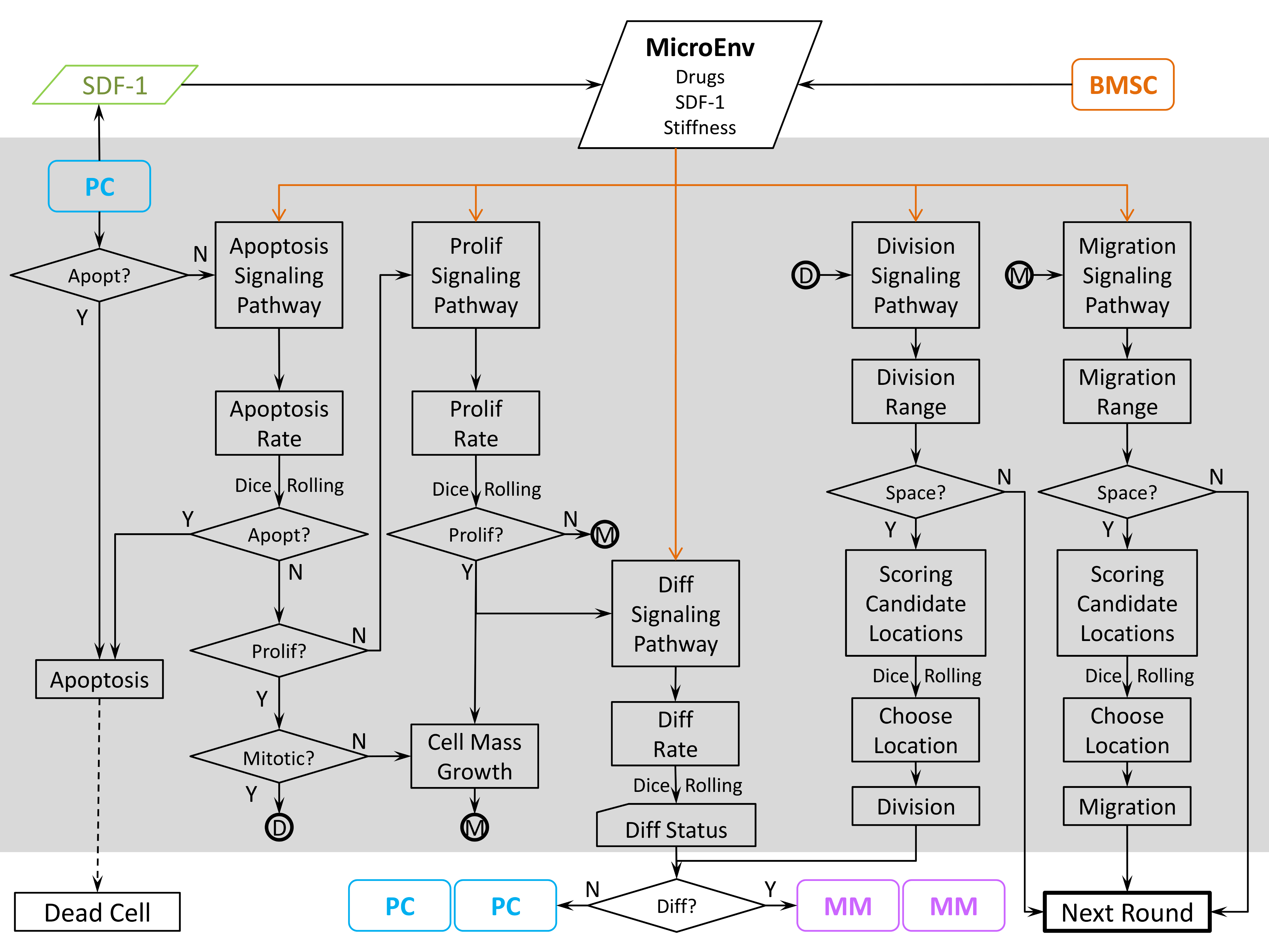

Supplement: Figure S3 — The flowchart of the PC agent. (TIF) [file pone.0085059.s003.tif]

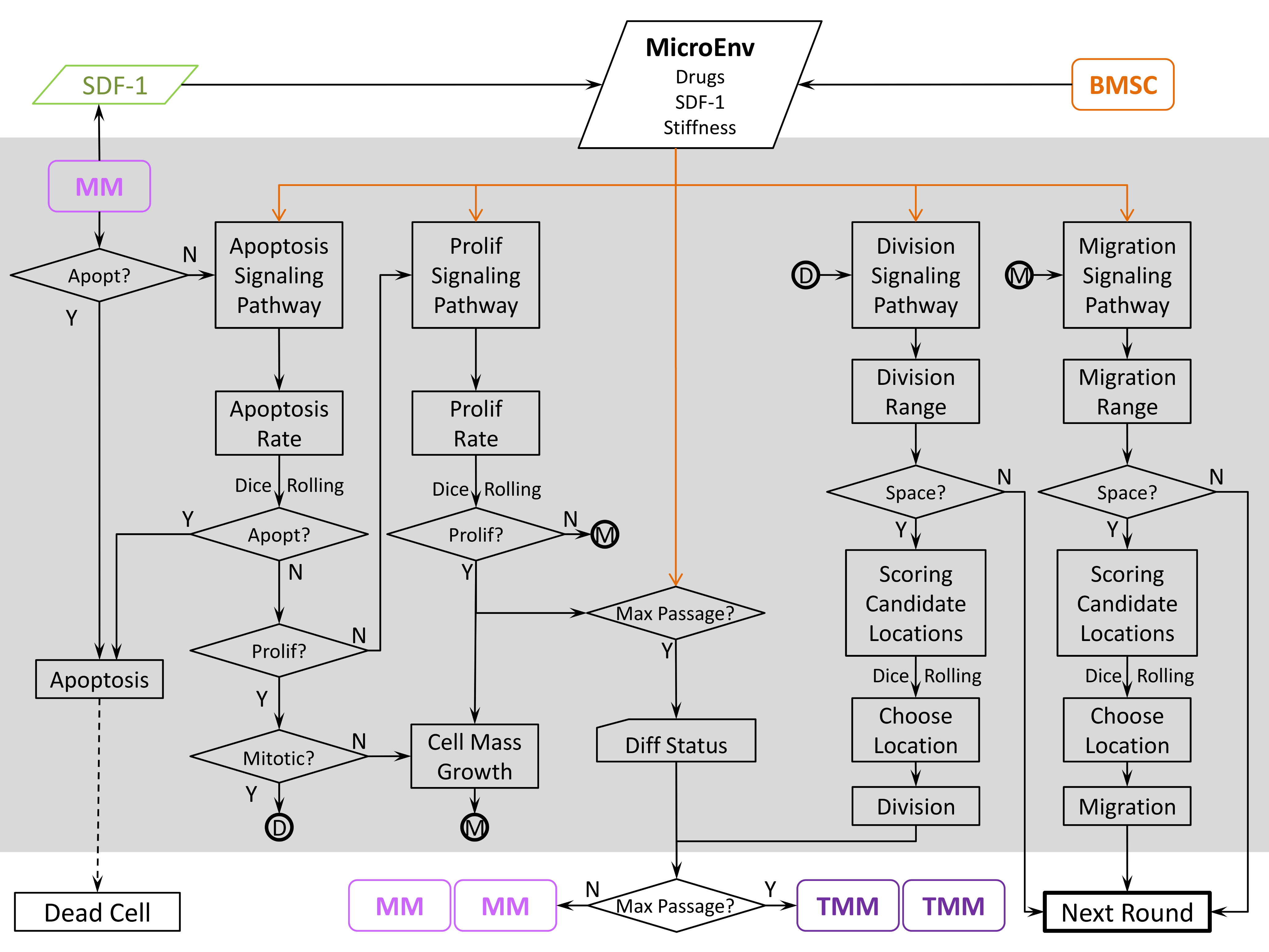

Supplement: Figure S4 — The flowchart of the MM agent. (TIF) [file pone.0085059.s004.tif]

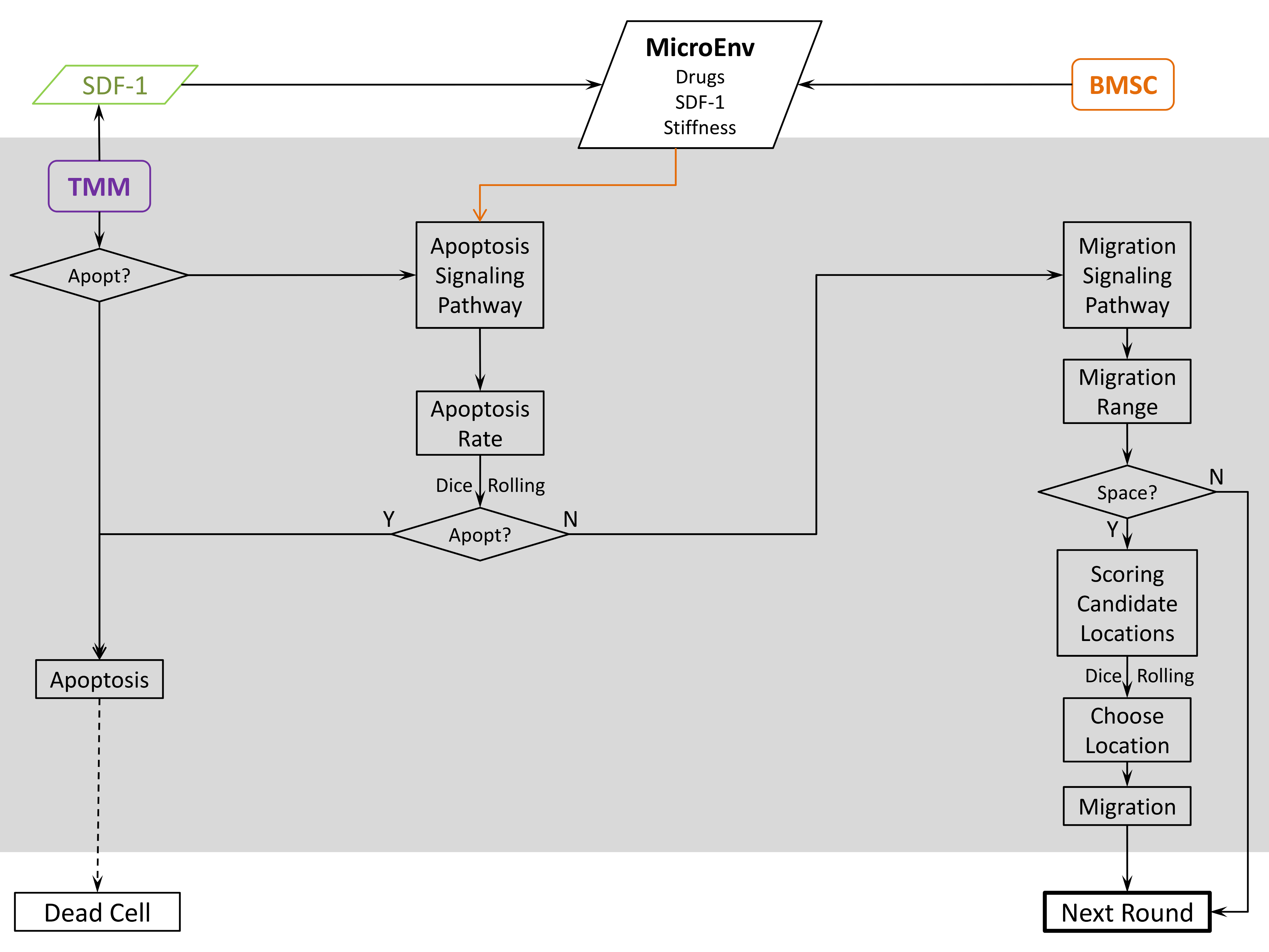

Supplement: Figure S5 — The flowchart of the TMM agent. (TIF) [file pone.0085059.s005.tif]
